# Genome sequence of the *Roseovarius mucosus* type strain (DSM 17069^T^), a bacteriochlorophyll *a*-containing representative of the marine *Roseobacter* group isolated from the dinoflagellate *Alexandrium ostenfeldii*

**DOI:** 10.1186/1944-3277-10-17

**Published:** 2015-03-19

**Authors:** Thomas Riedel, Stefan Spring, Anne Fiebig, Carmen Scheuner, Jörn Petersen, Markus Göker, Hans-Peter Klenk

**Affiliations:** 1 Leibniz Institute DSMZ – German Collection of Microorganisms and Cell Cultures, Inhoffenstraße 7B, 38124 Braunschweig, Germany; 2 Helmholtz Centre for Infection Research, Inhoffenstraße 7, 38124 Braunschweig, Germany

**Keywords:** Dinoflagellate, Plasmid, DMSP, Cytotoxin, Quorum sensing, Lithoheterotrophy, Photoheterotrophy, Mixotrophy, *Rhodobacteraceae*, *Alphaproteobacteria*

## Abstract

*Roseovarius mucosus* Biebl *et al*. 2005 is a bacteriochlorophyll *a*-producing representative of the marine *Roseobacter* group within the alphaproteobacterial family *Rhodobacteraceae*, which was isolated from the dinoflagellate *Alexandrium ostenfeldii*. The marine *Roseobacter* group was found to be abundant in the ocean and plays an important role for global and biogeochemical processes. Here we describe the features of the *R. mucosus* strain DFL-24^T^ together with its genome sequence and annotation generated from a culture of DSM 17069^T^. The 4,247,724 bp containing genome sequence encodes 4,194 protein-coding genes and 57 RNA genes. In addition to the presence of four plasmids, genome analysis revealed the presence of genes associated with host colonization, DMSP utilization, cytotoxins, and quorum sensing that could play a role in the interrelationship of *R. mucosus* with the dinoflagellate *A. ostenfeldii* and other marine organisms. Furthermore, the genome encodes genes associated with mixotrophic growth, where both reduced inorganic compounds for lithotrophic growth and a photoheterotrophic lifestyle using light as additional energy source could be used.

## Introduction

The *Roseobacter* group was shown to constitute a major component within the marine environments, encompassing around 20% of the bacterial community in coastal areas and 15% in mixed ocean-layers [1,2]. Strain DFL-24^T^ (= DSM 17069^T^ = NCIMB 14077^T^ = KACC 12996^T^) is the type strain of *R. mucosus*, originally isolated from a culture of the dinoflagellate *Alexandrium ostenfeldii* KO287, where it was found to be attached to the dinoflagellate surface [3]. *R. mucosus* DFL-24^T^ is one of currently seventeen species within the genus *Roseovarius* with a validly published name [4,5]. Whereas many *Roseovarius* species were named after their origin of isolation or their tolerances, the species epithet of strain DFL-24^T^ was chosen to reflect its slimy colony appearance [3].

Current PubMed records do not indicate any follow-up research with strain DFL-24^T^ after an overview about isolated aerobic anoxygenic phototrophs of the *Roseobacter* group from different marine habitats [6] and the initial description of *R. mucosus*[3]. Here, we analyzed the genome sequence of *R. mucosus* DSM 17069^T^. We present a description of the genome sequencing; its annotation and a summary classification together with a set of features for strain DFL-24^T^, including novel aspects of its phenotype.

### Organism information

#### ***Classification and features***

**Figure 1:**
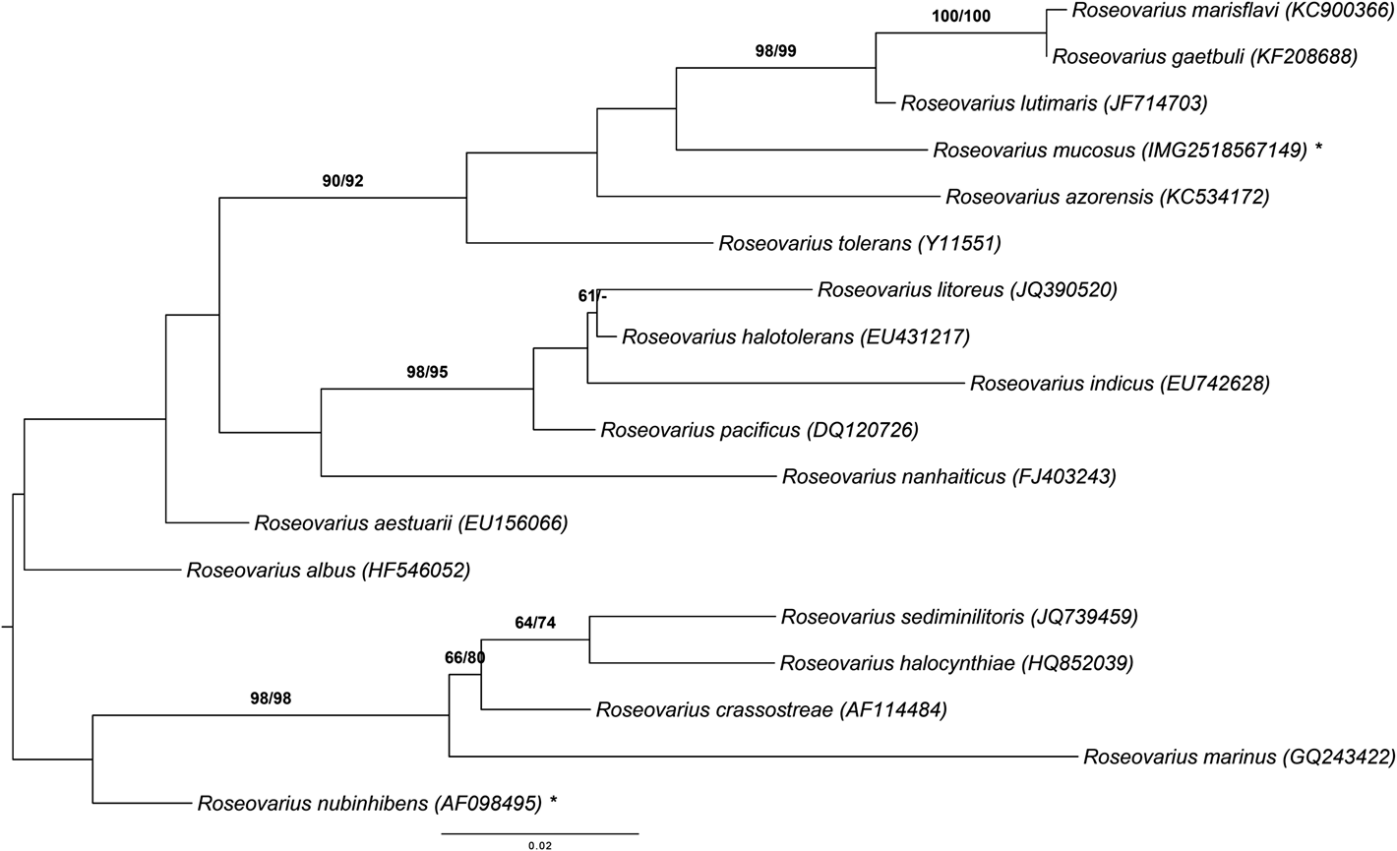
**Phylogenetic tree highlighting the position of *R. mucosus *relative to the type strains of the other species within the genus *Roseovarius*.** The tree was inferred from 1,386 aligned characters [7,8] of the 16S rRNA gene sequence under the maximum likelihood criterion [9]. Rooting was done initially using the midpoint method [10] and then checked for its agreement with the current classification (Table 1). The branches are scaled in terms of the expected number of substitutions per site. Numbers adjacent to the branches are support values from 1,000 ML bootstrap replicates [11] (left) and from 1,000 maximum-parsimony bootstrap replicates [12] (right) if larger than 60%. Lineages with type strain genome sequencing projects registered in GOLD [13] are labeled with one asterisk.

Figure 1 shows the phylogenetic neighborhood of *R. mucosus* in a 16S rRNA gene sequence based tree. The sequence of the single 16S rRNA gene copy in the genome is identical with the previously published 16S rRNA gene sequence (AJ534215).

The 16S rRNA gene sequence was compared using NCBI BLAST [14] under default settings (e.g., considering only the high-scoring segment pairs (HSPs) from the best 250 hits) with the most recent release of the Greengenes database [15] and the relative frequencies of taxa and keywords (reduced to their stem [16]) were determined, weighted by BLAST scores. The highest-scoring environmental sequence was HM591393 (Greengenes short name ‘Change microbial structure microfiltration and sand filter pretreatment systems seawater reverse osmosis process clone MF-July-41’), which showed an identity of 99.8% and a HSP coverage of 50.2%. The most frequently occurring keywords within the labels of all environmental samples which yielded hits were ‘microbi’ (3.4%), ‘lake’ (3.2%), ‘tin’ (2.9%), ‘xiaochaidan’ (2.7%) and ‘sediment’ (2.4%) (378 hits in total). The most frequently occurring keyword within the labels of those environmental samples which yielded hits of a higher score than the highest scoring species was ‘chang, filter, microbi, microfiltr, osmosi, pretreat, process, revers, sand, seawat, structur, system’ (8.3%) (1 hit in total). These keywords partially fit to the marine origin of strain DFL-24^T^.

**Figure 2:**
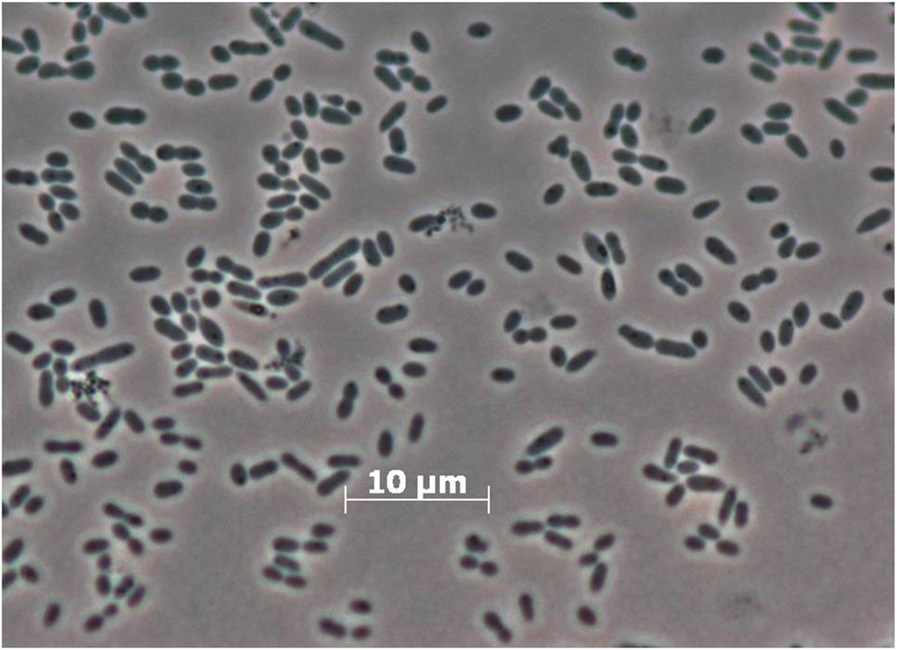
**Micrograph of strain *R. mucosus *** **DSM 17069**^**T**^**.**

Cells of strain DFL-24^T ^[3] are rod-shaped with characteristic pointed cell poles, 1.3-3.0 μm in length and 0.5-0.7 μm in width (Figure 2). Colonies on marine agar 2216 (BD Difco) are circular, convex and glistening, sometimes slimy, and white to slightly pinkish in color [3]. Growth occurs at a temperature range between 15 and 43°C, a pH range between 6.0 and 8.8 and a salinity between 1 and 7% sea salts [3]. Substrates for growth are acetate, butyrate, fumarate, glutamate, glycerol, lactate, pyruvate, malate and succinate; growth does not occur with citrate, glucose, fructose, ethanol and methanol [3]. Cells are positive for catalase and oxidase [3]. A summary of features can be found in Table 1.

**Table 1:** **Classification and general features of *R. mucosus *DFL-24**^**T **^**in accordance with the MIGS recommendations **[17] **published by the Genome Standards Consortium **[18]

**MIGS ID**	**Property**	**Term**	**Evidence code**
	Current classification	Domain *Bacteria*	TAS [19]
		Phylum *Proteobacteria*	TAS [20]
		Class *Alphaproteobacteria*	TAS [21,22]
		Order *Rhodobacterales*	TAS [22,23]
		Family *Rhodobacteraceae*	TAS [24]
		Genus *Roseovarius*	TAS [25]
		Species *Roseovarius mucosus*	TAS [3]
		Type strain DFL-24	TAS [3]
	Gram stain	Negative	TAS [3]
	Cell shape	Rod-shaped	TAS [3]
	Motility	Motile	NAS
	Sporulation	Non-sporulating	NAS
	Temperature range	15-43°C	TAS [3]
	Optimum temperature	31°C	TAS [3]
	Salinity range	0.3-10% (Sea salts)	TAS [3]
	Optimum Salinity	1-7% (Sea salts)	TAS [3]
	pH range	6.0-8.8	TAS [3]
	Optimum	7.5	TAS [3]
MIGS-22	Oxygen requirement	Aerobic	TAS [3]
	Carbon source	Complex, amino acids, sugars	TAS [3]
	Energy metabolism	Lithoheterotroph, photoheterotroph	NAS
MIGS-6	Habitat	Seawater, dinoflagellate-attached	TAS [3]
MIGS-15	Biotic relationship	Free-living	NAS
MIGS-14	Pathogenicity	None	NAS
	Biosafety level	1	TAS [26]
MIGS-23.1	Isolation	Culture of dinoflagellate *Alexandrium ostenfeldii*	TAS [3]
MIGS-4	Geographic location	Helgoland, Germany (North Sea)	TAS [3]
MIGS-5	Sample collection time	2002	NAS
MIGS-4.1 MIGS-4.2	Latitude – Longitude	54.196 – 7.893	NAS
MIGS-4.3	Depth	Not reported	
MIGS-4.4	Altitude	Not reported	

Evidence codes - TAS: Traceable Author Statement (i.e., a direct report exists in the literature); NAS: Non-traceable Author Statement (i.e., not directly observed for the living, isolated sample, but based on a generally accepted property for the species, or anecdotal evidence). Evidence codes are from of the Gene Ontology project [27]).

Strain *R. mucosus* DFL-24^T^ was described as showing no real motility [3]. However, in the course of this study cells were observed that show a characteristic movement conferred by flagella.

The utilization of carbon compounds by *R. mucosus* DSM 17069^T^ grown at 28°C was also determined for this study using Generation-III microplates in an OmniLog phenotyping device (BIOLOG Inc., Hayward, CA, USA). The microplates were inoculated with a cell suspension at a cell density of 95-96% turbidity and dye IF-A. Further additives were vitamin, micronutrient and sea-salt solutions. The plates were sealed with parafilm to avoid a loss of fluid. The exported measurement data were further analyzed with the opm package for R [28], using its functionality for statistically estimating parameters from the respiration curves such as the maximum height, and automatically translating these values into negative, ambiguous, and positive reactions. The reactions were recorded in three individual biological replicates.

According to the Generation-III plates, the strain was positive for pH 6, 1% NaCl, 4% NaCl, 8% NaCl, D-galactose, D-fucose, L-fucose, L-rhamnose, 1% sodium lactate, *myo*-inositol, glycerol, D-aspartic acid, troleandomycin, rifamycin SV, glycyl-L-proline, L-alanine, L-arginine, L-aspartic acid, L-glutamic acid, L-histidine, L-pyroglutamic acid, L-serine, lincomycin, niaproof, quinic acid, vancomycin, methyl-pyruvate, L-lactic acid, citric acid, *α*-keto-glutaric acid, D-malic acid, L-malic acid, nalidixic acid, lithium chloride, potassium tellurite, *γ*-amino-n-butyric acid, *β*-hydroxy-butyric acid, acetoacetic acid, propionic acid, acetic acid, sodium formate, butyric acid and the positive control.

The strain was negative for the negative control (i.e. a well without substrate), dextrin, D-maltose, D-trehalose, D-cellobiose, *β*-gentiobiose, sucrose, D-turanose, stachyose, pH 5, D-raffinose, *α*-D-lactose, D-melibiose, *β*-methyl-D-galactoside, D-salicin, *N*-acetyl-D-glucosamine, *N*-acetyl-*β*-D-mannosamine, *N*-acetyl-D-galactosamine, *N*-acetyl-neuraminic acid, D-glucose, D-mannose, D-fructose, 3-O-methyl-D-glucose, inosine, fusidic acid, D-serine, D-sorbitol, D-mannitol, D-arabitol, D-glucose-6-phosphate, D-fructose-6-phosphate, D-serine, minocycline, gelatin, guanidine hydrochloride, pectin, D-galacturonic acid, L-galactonic acid-*γ*-lactone, D-gluconic acid, D-glucuronic acid, glucuronamide, mucic acid, D-saccharic acid, tetrazolium violet, tetrazolium blue, *p*-hydroxy-phenylacetic acid, D-lactic acid methyl ester, bromo-succinic acid, tween 40, α-hydroxy-butyric acid, *α*-keto-butyric acid, aztreonam and sodium bromate.

As far as present on Generation-III microplates, all growth tests conducted by Biebl *et al. *[3] were confirmed, and a number of additional sugars and amino acids were shown to be utilized by *R. mucosus* DSM 17069^T^. In accordance with the growth results, *R. mucosus* DSM 17069^T^ prefers carboxylic acids as the main carbon sources in the respiratory measurements. In contrast to the results recorded in [3], the Phenotype Microarray reaction to citrate was positive. This may be due to respiratory measurements being more sensitive than growth measurements [29].

#### ***Chemotaxonomy***

The principal cellular fatty-acid composition of strain DFL-24^T^ was determined by Biebl *et al. *[3]. The major fatty acids (>10% of total) identified in strain DFL-24^T^ are C_18:1_*ω*7*c* and C_16:0_ and were found to be similar within the genus *Roseovarius* [25]. As predominant isoprenoid quinone ubiquinone-10 (Q-10) was found [3], which is typical for the genus *Roseovarius* [25] and the majority of the class *Alphaproteobacteria*.

### Genome sequencing and annotation

#### ***Genome project history***

The genome of *R. mucosus* DSM 17069^T^ was sequenced within the project “Ecology, Physiology and Molecular Biology of the *Roseobacter* clade: Towards a Systems Biology Understanding of a Globally Important Clade of Marine Bacteria”. The strain was chosen for genome sequencing according the *Genomic Encyclopedia of Bacteria and **Archaea* criteria [30,31].

Project information is stored at the Genomes OnLine Database [13]. The Whole Genome Shotgun (WGS) sequence was produced using state of the art sequencing technology [32] and can be found at GenBank and the Integrated Microbial Genomes database [33]. A summary of the project information is shown in Table 2.

**Table 2:** Genome sequencing project information

**MIGS ID**	**Property**	**Term**
MIGS-31	Finishing quality	Non-contiguous finished
MIGS-28	Libraries used	Illumina PE library (350 bp insert size), 454 PE library (3 kb insert size)
MIGS-29	Sequencing platforms	Illumina GA IIx, Illumina MiSeq, 454 GS-FLX + Titanium
MIGS-31.2	Fold coverage	191 ×
MIGS-30	Assemblers	Velvet version 1.1.36, Newbler version 2.3, Consed 20.0
MIGS-32	Gene calling method	Prodigal 1.4
	Locus Tag	rosmuc
	Genbank ID	AONH00000000
	Genbank Date of Release	February 5, 2014
	GOLD ID	Gi21385
	BIOPROJECT	188077
	Project relevance	Tree of Life, biodiversity
MIGS-13	Source material identifier	DSM 17069^T^

#### ***Growth conditions and DNA isolation***

A culture of strain DSM 17069^T^ was grown aerobically in DSMZ medium 514 [34] at 28°C. Genomic DNA was isolated using Jetflex Genomic DNA Purification Kit (GENOMED 600100) following the standard protocol provided by the manufacturer but modified by an incubation time of 60 min, incubation on ice overnight on a shaker, the use of additional 50 μl proteinase K, and the addition of 100 μl protein precipitation buffer. DNA is available from the DSMZ through the DNA Network [35].

#### ***Genome sequencing and assembly***

The genome was sequenced using a combination of two libraries (Table 2). Illumina sequencing was performed on a GA IIx platform with 150 cycles. The paired-end library contained inserts of an average insert size of 350 bp. The first run on an Illumina GAII platform delivered 3.0 million reads. A second Illumina run was performed on a Miseq platform to gain a higher sequencing depth. To achieve longer reads, the library was sequenced in one direction for 300 cycles, providing another 2.0 million reads. Error correction and clipping were performed by fastq-mcf [36] and quake [37]. The data was assembled using velvet [38]. The first draft assembly from 2,822,784 filtered reads with an average read length of 165 bp resulted in more than 120 unordered contigs.

To improve the assembly, an additional 454 run was performed. The paired-end jumping library of 3 kb insert size was sequenced on a 1/8 lane. Pyrosequencing resulted in 92,601 reads with an average read length of 371 bp assembled in Newbler (Roche Diagnostics).

Both draft assemblies (Illumina and 454 sequences) were fractionated into artificial Sanger reads of 1000 bp in length plus 75 bp overlap on each site. These artificial reads served as an input for the phred/phrap/consed package [39]. By manual editing the number of contigs could be reduced to 26, localized in 17 scaffolds. The combined sequences provided a 191 × coverage of the genome.

#### ***Genome annotation***

Genes were identified using Prodigal [40] as part of the JGI genome annotation pipeline. The predicted CDSs were translated and used to search the National Center for Biotechnology Information\nonredundant database, UniProt, TIGR-Fam, Pfam, PRIAM, KEGG, COG, and InterPro databases. Identification of RNA genes were carried out by using HMMER 3.0rc1 [41] (rRNAs) and tRNAscan-SE 1.23 [42] (tRNAs). Other non-coding genes were predicted using INFERNAL 1.0.2 [43]. Additional gene prediction analysis and functional annotation was performed within the Integrated Microbial Genomes - Expert Review platform [44]. CRISPR elements were detected using CRT [45] and PILER-CR [46]. The annotation on the IMG-ER platform was used for the genome analysis and its genome description; the annotation of the NCBI deposit of the genome as well as later version on IMG might slightly differ from the figures given below.

#### ***Genome properties***

**Figure 3:**
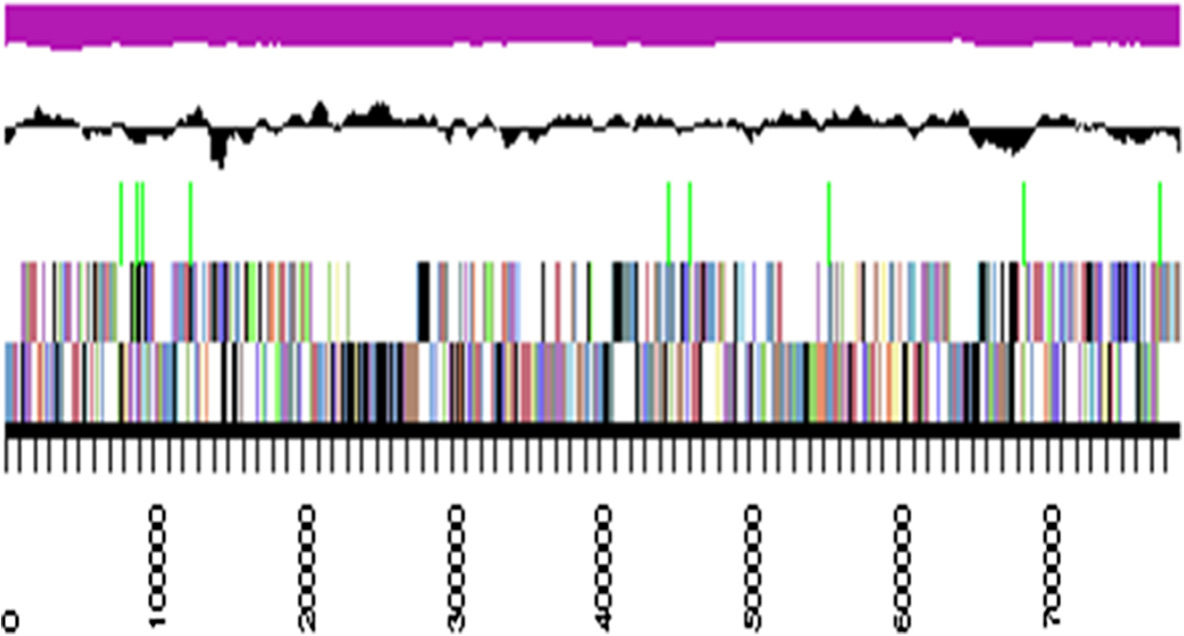
**Graphical map of the largest scaffold.** From bottom to the top: Genes on forward strand (colored by COG categories), Genes on reverse strand (colored by COG categories), RNA genes (tRNAs green, rRNAs red, other RNAs black), GC content (black), GC skew (purple/olive).

The genome statistics are provided in Table 3 and Figure 3. The genome of strain DSM 17069^T^ has a total length of 4,247,724 bp and a G + C content of 61.9%. Of the 4,251 genes predicted, 4,194 were identified as protein-coding genes, and 57 as RNAs. The majority of the protein-coding genes were assigned a putative function (81.6%) while the remaining ones were annotated as hypothetical proteins. The distribution of genes into COGs functional categories is presented in Table 4.

**Table 3:** Genome statistics*

**Attribute**	**Value**	**% of total**
Genome size (bp)	4,247,724	100.00
DNA coding region (bp)	3,882,232	91.40
DNA G + C content (bp)	2,629,252	61.90
DNA scaffolds	17	
Extrachromosomal elements	4	
Total genes	4,251	100.00
RNA genes	57	1.34
rRNA operons	1	
tRNA genes	43	1.01
Protein-coding genes	4,194	98.66
Genes with function prediction (proteins)	3,467	81.56
Genes in paralog clusters	1,438	33.83
Genes assigned to COGs	3,319	78.08
Genes assigned Pfam domains	3,538	83,23
Genes with signal peptides	371	8.73
Genes with transmembrane helices	938	22.07
CRISPR repeats	0	

*The annotation on IMG [33,44] is subject to regular updates; the numbers presented here might deviate from a later version of the genome.

**Table 4:** Number of genes associated with the general COG functional categories*

**Code**	**value**	**% age**	**Description**
J	166	4.5	Translation, ribosomal structure and biogenesis
A	0	0.0	RNA processing and modification
K	262	7.2	Transcription
L	146	4.0	Replication, recombination and repair
B	4	0.1	Chromatin structure and dynamics
D	35	1.0	Cell cycle control, cell division, chromosome partitioning
Y	0	0.0	Nuclear structure
V	49	1.3	Defense mechanisms
T	149	4.1	Signal transduction mechanisms
M	189	5.2	Cell wall/membrane/envelope biogenesis
N	87	2.4	Cell motility
Z	1	0.0	Cytoskeleton
W	0	0.0	Extracellular structures
U	102	2.8	Intracellular trafficking and secretion, and vesicular transport
O	161	4.4	Posttranslational modification, protein turnover, chaperones
C	253	6.9	Energy production and conversion
G	147	4.0	Carbohydrate transport and metabolism
E	442	12.1	Amino acid transport and metabolism
F	70	1.9	Nucleotide transport and metabolism
H	173	4.7	Coenzyme transport and metabolism
I	146	4.0	Lipid transport and metabolism
P	177	4.8	Inorganic ion transport and metabolism
Q	126	3.5	Secondary metabolites biosynthesis, transport and catabolism
R	431	11.8	General function prediction only
S	339	9.3	Function unknown
-	932	21.9	Not in COGs

*The annotation on IMG [33,44] is subject to regular updates; the numbers presented here might deviate from a later version of the genome.

### Insights from the genome sequence

#### ***Genome comparisons and extrachromosomal elements***

The two other *Roseovarius* genomes, *Roseovarius nubinhibens* ISM (IMG taxon ID 638341183 = AALY00000000; total number of genes: 3605) and *Roseovarius* sp. TM1035 (IMG taxon ID 640963035 = ABCL00000000; total number of genes: 4158), both reveal a genome length shorter than that of strain DSM 17069^T^, but also remain still in draft state.

**Figure 4:**
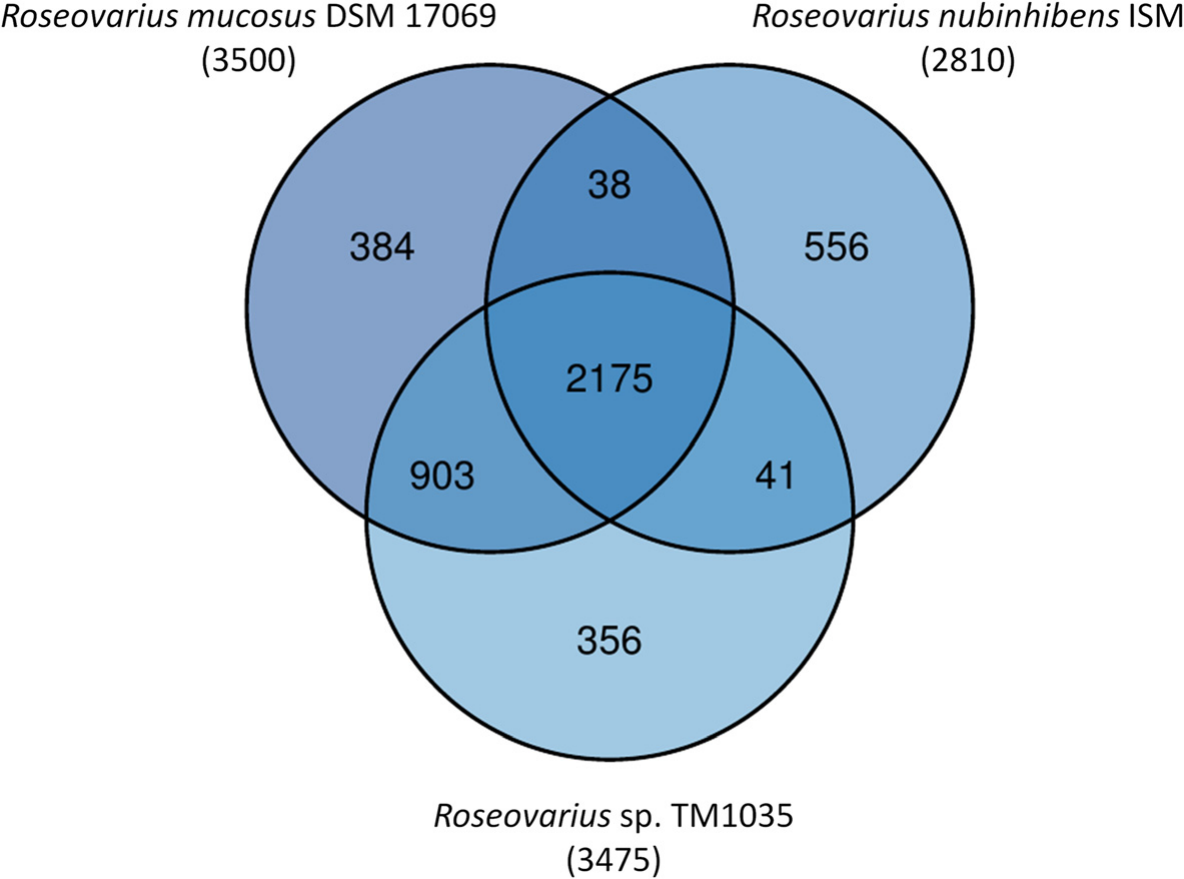
**Venn diagram depicting the intersections of sets of homologous proteins of *R. mucosus *DSM 17069**^**T**^**, *R. nubinhibens *ISM and *Roseovarius *sp. TM1035.** Their cardinalities are given in parentheses; the total number of proteins and the resources are listed in the text. The Venn diagram was calculated with the corresponding R package [47].

The fraction of shared genes between strain *R. mucosus* DSM 17069^T^ and the strains *R. nubinhibens* ISM and *Roseovarius* sp. TM1035 is shown in a Venn diagram (Figure 4). The numbers of pairwise shared homologous genes were inferred from an analysis with a reimplementation of the TribeMCL algorithm [48], applying an E-value threshold of 10^-5^ and an MCL inflation parameter of 2.0.

Many members of the *Roseobacter* group contain plasmids [49]. Genome sequencing of strain *R. mucosus* DSM 17069^T^ revealed the presence of four plasmids with sizes between 21 kb and 123 kb (Table 5). The two largest plasmids pRosmuc_A123 and pRosmuc_B85 contain characteristic RepABC replication operons including the *repAB* partitioning genes. The respective replicases that mediate the initiation of replication are designated according to the established plasmid classification scheme [50]. The different numbering of the replicases RepC-7 and RepC-1 corresponds to specific plasmid compatibility groups that are required for a stable coexistence of the replicons within the same cell. The smallest plasmid pRosmuc_D21 contains a RepAIV type replicase [51], but a typical *parAB* partitioning operon is lacking.

**Table 5:** **General genomic features of the chromosome and extrachromosomal replicons from *R. mucosus *** **strain DSM 17069**^**T**^

**Replicon**	**Contig**	**Replicase**	**Length (bp)**	**GC (%)**	**Topology**	**No. genes**^**#**^
Chromosome^1^	283	DnaA	627 421	61	linear*	625
pRosmuc_A123	58	RepC-7 RepA-III	123 088	59	linear*	141
pRosmuc_B85	111	RepC-1	85 327	60	linear*	85
pRosmuc_C27	46	RepA-?	26 795	64	linear*	30
pRosmuc_D21	45	RepA-IV	20 721	57	linear*	24

*Circularity not experimentally validated; ^#^deduced from automatic annotation; ^1^partial sequence including the replicase *dnaA* (rosmuc_00382).

This distribution may correspond with a higher plasmid copy number within the cell thus assuring the replicon maintenance in the daughter cells after cell division. The plasmid pRosmuc_C27 harbors a ParF-type ATPase (rosmuc_03930) downstream of the replicase RepA, which may ensure successful partitioning. The largest plasmid pRosmuc_A123 contains apart from the RepC-7 replication operon an additional solitary replicase RepA-III and may hence represent a composite replicon resulting from plasmid fusion [52].

The locus tags of all replicases, plasmid stability modules and the large *virB4* and *virD4* genes of type IV secretion systems are presented in Table 6. The 85 kb plasmid pRosmuc_B85 contains two postsegregational killing systems (PSK) consisting of a typical operon with two small genes encoding a stable toxin and an unstable antitoxin [53]. The RepABC-7/RepA-III-type plasmid pRosmuc_A123 contains a complete type-IV secretion system including the *virB* operon for the formation of a transmembrane channel. The relaxase VirD2, which is required for the strand-specific DNA nicking at the origin of transfer (*ori*T), and the coupling protein VirD4 support the presence of functional conjugation systems [49,54]. Moreover, this plasmid contains an additional MobA-type relaxase (rosmuc_04055) located in close proximity to a second coupling protein VirD4 (rosmuc_04058) and the replicase RepA-III (rosmuc_04060) thus supporting the idea that the replicon represents a composite plasmid.

**Table 6:** **Integrated Microbial Genome (IMG) locus tags of *R. mucosus *** **DSM 17069**^**T **^**genes for the initiation of replication, toxin/antitoxin modules and two representatives of type IV secretion systems (T4SS) that are required for conjugation**

**Replicon**	**Replication initiation**	**Plasmid stability**	**Type IV secretion**			
	**Replicase**	**Locus tag**	**Toxin**	**Antitoxin**	**VirB4**	**VirD4**
Chromosome	DnaA	rosmuc_00382	-	-	-	-
pRosmuc_A123	RepC-7, RepA-III	rosmuc_04171, rosmuc_04060	-	-	rosmuc_04111	rosmuc_04128, rosmuc_04038
pRosmuc_B85	RepC-1	rosmuc_04219	rosmuc_04223, rosmuc_04227	rosmuc_04222, rosmuc_04226	-	-
pRosmuc_C27	RepA-?	rosmuc_03931	-	-	rosmuc_03913	rosmuc_03918
pRosmuc_D21	RepA-IV	rosmuc_03891	-	-	-	rosmuc_03890

#### ***Commensalism and pathogenic potential***

Commensal bacteria colonize a host and benefit from it without having a negative effect on its growth. Several representatives of the *Roseobacter* group were found to live in a close and stable ecological relationship with phototrophic dinoflagellates [2]. This symbiosis is not obligate, so that both organisms can also proliferate independently from each other. The exact type of the association between dinoflagellates and *Roseobacter* species is, however, rarely determined and may vary depending on the involved species. In the case of *Dinoroseobacter shibae* a mutualistic symbiosis was found in which the dinoflagellate assimilates essential B vitamins that are produced by the bacterium [55]. In the following paragraphs some features encoded in the genome of strain DSM 17069^T^ are mentioned that could play a role in the interrelationship of *R. mucosus* with the dinoflagellate *A. ostenfeldii* and other marine organisms.

The first step in establishing a stable association of free-living *R. mucosus* cells with a potential dinoflagellate host is the attachment to its surface. Factors that could control attachment are the production of flagella and pili. Swimming cells were rarely observed in laboratory cultures of strain DSM 17069^T^ (this study), but may play an important role in the marine ecosystem for targeting dinoflagellates for subsequent colonizing. Genes for the synthesis of flagellae, a flagellar motor and a chemotactic response were located dispersed within the genome sequence, but the two largest gene clusters are found at rosmuc_00543-00574 (mainly genes for the flagellar basal body) and rosmuc_02499-02544 (flagellar motor and chemotaxis). After reaching a suitable dinoflagellate, pili or fimbriae could mediate attachment to the surface of the eukaryotic host. Genes encoding the synthesis of Flp pili belonging to the type IV family were located at rosmuc_01726-01737. It is assumed that Flp pili play a role in the attachment of bacteria to eukaryotic cells, and it was reported that in *Ralstonia solanacearum* Flp pili are required for the virulence on potato [56]. Upon successful attachment bacteria colonizing surfaces often produce extracellular polymeric substances to establish biofilms that stabilize the association and provide a suitable microenvironment for growth. Formation of slime capsules by cells of *R. mucosus* is suggested by a slimy appearance of colonies on agar plates [3]. In addition, the genome of DSM 17069^T^ encodes two transporters for the export of capsular polysaccharides, one is of the ABC type (rosmuc_00889-00891) and the other belongs to the CPS-E family (rosmuc_01918-01920).

Dimethylsulfoniopropionate (DMSP) is a common metabolite of marine phytoplankton, especially phototrophic dinoflagellates. It is a compatible solute that can reach high intracellular concentrations and probably has various cellular functions, but osmoregulation seems to be the most important one [57]. Intracellular DMSP can be released into the environment by leakage, senescence or lysis and represents therefore a major source of reduced carbon and sulfur for heterotrophic marine bacteria. Due to the rapid dilution of DMSP in the marine surface water, bacteria colonizing DMSP producers have an advantage over free-living bacteria. DMSP can be utilized as substrate either by cleavage or demethylation pathways, and *R. mucosus* seems to encode key genes of both routes. In the genome of DSM 17069^T^ genes for a dimethylsulfoniopropionate demethylase (DmdA) were located at two different sites (rosmuc_01718 and rosmuc_02463), which were both reliably identified by sharing protein-sequence identity values above 85% with translated *dmdA* genes of other *Roseovarius* strains. The DmdA enzyme catalyzes the transfer of a methyl group to tetrahydrofolate (THF) resulting in 5-methyl-THF and methylmercaptopropionate (MMPA) [58]. The DmdA gene at rosmuc_01718 is probably part of an operon that encodes also the genes for acyl-CoA synthetase (DmdB, rosmuc_01719), methylmercaptopropionyl-CoA dehydrogenase (DmdC, rosmuc_01721) and methylthioacryloyl-CoA hydratase (DmdD, rosmuc_01720), which catalyze the further degradation of MMPA to acetaldehyde and methanethiol. Acetaldehyde and 5-methyl-THF can be funneled into the central carbon metabolism, whereas the reduced sulfur of methanethiol is probably assimilated as methionine by the enzyme cystathionine gamma-synthetase (rosmuc_01420). The alternative DMSP cleavage pathway starts in *R. mucosus* with the dimethylsulfoniopropionate lyase DddP (rosmuc_03355) that was originally identified in *R. nubinhibens* and produces dimethylsulfide (DMS) and acrylate from DMSP. No genes encoding enzymes acting on DMS were identified in the genome of DSM 17069^T^, so that this volatile sulfur compound cannot be further utilized by *R. mucosus* and is released into the environment. Consequently, the DMSP cleavage pathway is probably mainly used if enough energy for the sulfur assimilation by the reduction of sulfate via the APS/PAPS pathway is available.

The genome of *R. mucosus* encodes a large number of potentially cytotoxic compounds, including more than 20 genes encoding RTX-like toxins. The cytolytic activity of RTX toxins is based on forming cation-selective pores in membranes of target cells which eventually leads to cell death and lysis [59]. Typical RTX-like toxins are post-translationally activated by fatty acylation and exported into the extracellular space by a unique type I secretion system that mediates translocation across the cytoplasmic and outer membrane in a single step. Albeit some of the RTX protein genes in *R. mucosus* DSM 17069^T^ were annotated as putative hemolysins (e.g., rosmuc_00250, rosmuc_00896, rosmuc_01947, and rosmuc_02953), they were not located in typical RTX operons encoding also genes for a type I secretion system and a specific acyltransferase. Apparently, these genes are dispersed within the genome and likely expressed constitutively. For instance, genes for a complete type-I secretion system comprising an outer membrane protein, ABC transporter and the membrane fusion protein are encoded at rosmuc_03175-03177. Although, RTX-like toxins are secreted from some representatives of the *Roseobacter* group in large amounts [60], their ecological function in avirulent strains is largely unknown. Potential functions could include the inhibition of competing bacterial species colonizing the dinoflagellate surface, a contribution to the killing or incapacitation of zooplankton species grazing on the dinoflagellate host or an infection of shellfish after filter feeding on dinoflagellates. In this regard it is noteworthy that the related species *Roseovarius crassostreae* was identified as a putative pathogen of juvenile oysters [61]. Alternative functions of RTX-like proteins could, however, also include various exo-enzymatic activities that may be required for the release of dissolved organic carbon from macromolecules [59].

Many representatives of the *Roseobacter* group encode and express gene sequences associated with quorum sensing (QS), a process of cell-to-cell communication and interaction between relatives in dependence of a certain detected population threshold value [62]. They produce small signal molecules called autoinducers, which are detected by their conspecific bacteria when excreted in the environment. Consequently, when the concentration of those membrane-diffusible autoinducers reaches a specific threshold value, the population responds with an activation of gene expression to coordinate a population-wide behavior. Especially, for bacteria colonizing or infecting hosts it is important to determine if the cell density of their population is high enough to make colonization or infection successful. Genome analysis of *R. mucosus* DSM 17069^T^ revealed the presence of genes putatively associated with QS activity, like e.g. a N-acyl-L-homoserine lactone synthetase (*luxI* homolog; rosmuc_03495) and several regulator proteins belonging to the *luxR* family (e.g., rosmuc_01010, rosmuc_02138, rosmuc_02881, rosmuc_03496, and rosmuc_03642).

#### ***Mixotrophic growth***

In the absence of a suitable dinoflagellate host *R. mucosus* has to survive as free-living cells in seawater. A well-known characteristic of most marine environments is a depletion of organic nutrients, so that the utilization of alternative energy sources is an important part of bacterial survival strategies in marine surface waters. In evolution marine heterotrophic bacteria have developed essentially two different approaches to overcome the lack of organic carbon sources as electron donors. One is the use of reduced inorganic compounds for lithoheterotrophic growth and the other harvesting of light as additional energy source for photoheterotrophy. According to the genome sequence both mechanisms for generation of energy could be operative in *R. mucosus* DSM 17069^T^.

A large cluster of genes involved in the production of a [NiFe] uptake-type hydrogenase (hydrogen:quinone oxidoreductase) was detected at rosmuc_03325-03344. A major source of hydrogen in surface seawater may be nitrogen-fixing cyanobacteria, where it is produced as by-product to the formation of ammonia from the nitrogenase enzyme [63].

Another potential electron donor in ocean waters is carbon monoxide (CO) that is formed by photolysis of dissolved organic matter [64]. It has been reported that only representatives of the *Roseobacter* group that encode both the definitive form I and a putative form II of the carbon monoxide dehydrogenase are able to oxidize CO to CO_2_ in laboratory experiments [65]. *R. mucosus* DSM 17069^T^ encodes indeed a carbon monoxide dehydrogenase of form I (*coxLSM*; rosmuc_00985-00987) and form II (*coxSLM*; rosmuc_01269-01271), so that this strain most likely can use CO as supplementary electron donor.

Besides the dissolved gasses H_2_ and CO reduced inorganic sulfur compounds represent main sources for mixotrophic growth in marine environments. Thiosulfate is one of the most common forms of reduced inorganic sulfur in seawater, because it is quite stable under oxic conditions and thus can accumulate to recognizable amounts in open ocean waters. It can be produced abiotically during oxidation of sulfide at oxic-anoxic transition zones or as metabolic by-product during the degradation of organic sulfur compounds. Especially, the latter source seems to be important at niches which are far away from sites of sulfide production but close to phytoplankton producing organic sulfur compounds. Examples of thiosulfate producing marine bacteria are *Chromatocurvus halotolerans* growing on glutathione [66] and *Methylophaga sulfidovorans* growing on dimethylsulfide [67]. However, in most aerobic marine bacteria the sulfur moiety of organic compounds seems to be oxidized to sulfate as end product, although it is assumed that thiosulfate may represent an intermediate metabolite in sulfur oxidation in some of these bacteria [66]. In most representatives of the *Roseobacter* group oxidation of thiosulfate to sulfate is catalyzed by the periplasmic Sox multienzyme complex, which allows the generation of additional energy for mixotrophic growth in *Ruegeria* (formerly *Sillicibacter*) *pomeroyi *[68]. In the genome of *R. mucosus* DSM 17069^T^ a large set of *sox* genes (*soxRSVWXYZABCDEF*) encoding all proteins of a functional Sox multienzyme complex was found at rosmuc_00222-00234. The generation of metabolically useful energy from the oxidation of thiosulfate was however not tested in this strain so far.

**Figure 5:**
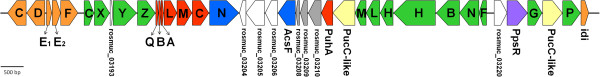
**Arrangement of the photosynthetic gene cluster.** Green, *bch* genes; red, *puf* genes; orange, *crt* genes; blue, *hem* genes; purple, genes for sensor proteins, white, other genes (adapted after [71,72]).

Light can be used as additional energy source by aerobic marine bacteria either by using rhodopsins or a photosynthetic apparatus. Genomic analyses of bacteria inhabiting the photic zone of marine environments revealed that harvesting of light using proton-pumping proteorhodopsins is widely distributed and frequently found in members of the *Flavobacteria**,**Alphaproteobacteria* and *Gammaproteobacteria* [69]. In contrast, bacteria expressing a photosynthetic apparatus enabling aerobic photoheterotrophic growth are mainly restricted to representatives of the *Roseobacter* group [2] and the gammaproteobacterial OM60/NOR5 clade [70]. A large coherent photosynthetic gene cluster comprising genes for bacteriochlorophyll and carotenoid synthesis, a photosynthetic reaction center and a light-harvesting antenna complex was detected in the genome of *R. mucosus* DSM 17069^T^ at rosmuc_03187-03225 (Figure 5). In addition, two proteins containing a BLUF domain enabling sensing of blue light using FAD were located outside of the photosynthesis gene cluster at rosmuc_00390 and rosmuc_04258. The description of *R. mucosus* reports low levels of cellular bacteriochlorophyll *a *[3], which indicates that the photosynthesis genes are expressed and functional in this strain. The regulation of photosynthetic gene expression was not further analyzed, so that it is unknown if higher amounts of bacteriochlorophyll *a* may be produced in cells of *R. mucosus* under distinct environmental conditions.

## Conclusion

Members of the marine *Roseobacter* group are widely distributed in the marine environment. In this study we analysed the genome sequence of *R. mucosus *DSM 17069^T^, which was isolated from the dinoflagellate *Alexandrium ostenfeldii*. Genome analysis of this type strain revealed the presence of key functional characteristics. We summarized some of them, such as genes associated with host colonization, DSMP utilization, cytotoxins and quorum sensing that could play a role in a possible interrelationship of *R. mucosus* with the dinoflagellate *A. ostenfeldii* and other marine organisms. In addition, genome analysis of *R. mucosus* DSM 17069^T^ revealed a possible lithotrophic as well as photoheterotrophic lifestyle.

## Abbreviations

HSP: High-scoring Segment Pair; ML: Maximum Likelihood; WGS: Whole Genome Shotgun; MCL: Markov Cluster; DMSP: Dimethylsulfoniopropionate; DmdA: Dimethylsulfoniopropionate demethylase; DmdB: Acyl-CoA synthetase; DmdC: Methylmercaptopropionyl-CoA dehydrogenase; DmdD: Methylthioacryloyl-CoA hydratase; DddP: Dimethylsulfoniopropionate lyase; THF: Tetrahydrofolate; MMPA: Methylmercaptopropionate; DMS: Dimethylsulfide; QS: Quorum Sensing; BLUF: Sensors of Blue Light using FAD; FAD: Flavin Adenine Dinucleotide.

## Competing interests

The authors declare that they have no competing interests.

## Authors’ contributions

MG and HPK devised the study. TR, SS, AF, CS, JP, MG, and HPK contributed materials and analyses. TR wrote the paper. All authors read and approved the final manuscript.
